# Clearance Criteria for Determining Eligibility for Force Plate Testing After Anterior Cruciate Ligament Reconstruction: A Scoping Review

**DOI:** 10.3390/medicina62030503

**Published:** 2026-03-09

**Authors:** Landon Christoffel, Lauren Beaupre, Stephanie Nathanail, Wasim Labban, Mark Sommerfeldt, Lindsey Westover, Gail M. Thornton

**Affiliations:** 1 Department of Physical Therapy, University of Alberta, Edmonton, AB T6G 2R3, Canada; lauren.beaupre@ualberta.ca (L.B.);; 2 Department of Surgery, University of Alberta, Edmonton, AB T6G 2R3, Canada; 3 Alberta Health Services, Edmonton, AB T6G 2B7, Canada; 4 Mirdif Center for Physiotherapy and Rehabilitation, Dubai P.O. Box 119590, United Arab Emirates; 5 Department of Mechanical Engineering, University of Alberta, Edmonton, AB T6G 2R3, Canadagail.thornton@ualberta.ca (G.M.T.); 6 Department of Biomedical Engineering, University of Alberta, Edmonton, AB T6G 2R3, Canada; 7 Health Technology & Policy Unit, University of Alberta, Edmonton, AB T6G 2R3, Canada

**Keywords:** anterior cruciate ligament, anterior cruciate ligament reconstruction, force plate testing, return to play, limb symmetry

## Abstract

*Background and Objectives*: Throughout the return-to-play process after anterior cruciate ligament reconstruction (ACLR), clearance criteria and limb symmetry indices (LSI) play an important role in clinical decision-making by helping evaluate patient readiness and informing safe activity progressions, with the goal of reducing re-injury risk. How clearance criteria are implemented in research studies to evaluate patient readiness, specifically in force plate jumping studies, is currently unknown. This scoping review was a focused examination of clearance criteria and limb symmetry indices in studies performing force plate-based jumping assessments with ACLR patients. The research questions guiding this scoping review were as follows: (1) What clearance criteria are reported in studies involving primary ACLR patients who participate in jumping assessments on force plates? (2) What LSI are reported in force plate studies, and what level of symmetry is deemed acceptable to allow for safe participation of ACLR patients who participate in jumping assessments of force plates? *Materials and Methods*: Nine databases were searched on 7 or 8 September 2024 for three concepts: ACLR, force plates, and movement properties. Inclusion criteria were as follows: (a) primary ACLR patients at least 6 months post-surgery; (b) performing a countermovement or drop jump; (c) collecting at least one kinetic parameter using a force plate. Clearance criteria was operationally defined as a time from surgery boundary, functional or performance-based testing criteria, medical evaluation, or completion/participation in a rehabilitation program. *Results*: Thirty-five studies were included. Time from surgery was the most frequently reported clearance criteria (26/35; 74.3%), followed by medical evaluation (18/35; 51.4%), and completion of rehabilitation (10/35; 28.6%). Use of LSI as clearance criteria was limited (5/35; 14.3%). Minimum required LSI ranged from 85 to 90% in quadriceps strength and hop testing. *Conclusions*: Clearance criteria varied by jump type and post-surgical time frame when the participant was tested. Standardized rehabilitation was common prior to 2 years post-surgery, whereas medical clearance was common after 2 years post-surgery. Single leg jumps typically required 2–3 clearance criteria, whereas double leg jumps required 1–2 clearance criteria. Limb symmetry indices were used in combination with two other clearance criteria in studies with single-leg countermovement or drop jumps. Improvements in clearance criteria and adverse event reporting may help improve patient safety and interpretation of findings across studies.

## 1. Introduction

Global incidence rates of anterior cruciate ligament (ACL) reconstructions (ACLR) continue to climb each year across all levels of sport in both females and males, with data from Australia, Canada and Italy reporting increases in ACLR injury rates of 0.96–6.2% per year between the 1990s and late 2010s [1,2,3]. Overall, the clinical community has begun to shift from solely time-based rehabilitation and return-to-play (RTP) protocols in favor of criterion-based rehabilitation progressions with the hope of better long-term outcomes following RTP [4,5,6,7,8,9,10]. Rehabilitation progression varies by athlete and practitioner; however, overall goals of the rehabilitation process include restoring range of motion (ROM), eliminating pain and swelling, restoring strength and postural stability, and regaining any specific physical performance qualities necessary for the athlete’s sport [4,5,6,7,8,9,10]. Additional recommendations for clearance criteria for RTP include ligament stability during clinical examination, limb symmetry indices (LSI) of at least 85–90% on single-leg strength and hop tests, and adequate psychological readiness [4,5,6,7,8,9,10].

In clinical practice, a patient often needs to exhibit adequate strength and neuromuscular control before initiating dynamic activities like jumping and cutting [5,6,9,10,11]. Jumping tasks are considered a high-risk movement post-ACLR due to high ground reaction forces exerted on the knee, requirements for rapid engagement of the lower limb musculature, and propensity for poor landing mechanics by ACLR patients, which can all place high degrees of stress on the graft [12,13,14,15,16,17,18,19,20]. An increasingly popular measurement tool in both clinical practice and research settings is a dual force plate system. Dual force plates can be used by researchers and clinicians alike to evaluate lower limb health by analyzing kinetic parameters and neuromuscular deficits between limbs such as rate of force development and impulse asymmetries that may be exhibited during jumping tasks following ACLR. A previous systematic review and meta-analysis by our research group (Labban et al., 2024) examined two commonly used jump types (countermovement jump [CMJ] and drop jump [DJ]) and the kinetic parameters reported in force plate studies involving the ACLR population and how these individuals differ in performance relative to healthy controls [21].

In ACLR studies, participants may be recruited from the community, without the research team having direct knowledge of the rehabilitation program followed by the participant. Thus, similar to criterion-based progressions in clinical settings, it may be advisable for clearance criteria to be implemented in studies involving force plate jumping in ACLR patients to help ensure adequate readiness for jump testing. Hypothetically, this may reduce risk of harm for the participant and provide researchers and clinicians with more objective information regarding participants’ physical readiness at time of testing, improving the generalizability of the study findings to other research and/or clinical contexts. Many reviews have examined how clinicians assess readiness for RTP using clearance criteria [5,6,7,8,9,10,22,23,24]; however, currently, no known studies have investigated the reporting of clearance criteria used with ACLR patients to determine their readiness to perform jump testing in force plate studies. This scoping review was a focused examination of clearance criteria and limb symmetry indices in studies performing force plate-based jumping assessments with ACLR patients.

## 2. Methods

### 2.1. Registration

This scoping review was prospectively registered on Open Science Framework Registries https://doi.org/10.17605/OSF.IO/E9TVN [25].

### 2.2. Framework

This scoping review used the framework proposed by Arksey & O’Malley (2005) [26] and enhanced by Levac et al. (2010) [27] and was reported using Preferred Reporting Items for Systematic Review and Meta-Analyses extension for Scoping Reviews (PRISMA-ScR) guidelines [28].

### 2.3. Identifying Research Questions

The research questions were as follows: (1) What clearance criteria are reported in studies involving primary ACLR patients who participate in jumping assessments on force plates? (2) What LSI are reported in force plate studies, and what level of symmetry is deemed acceptable to allow for safe participation of ACLR patients who participate in jumping assessments of force plates?

### 2.4. Identifying Relevant Studies

With the assistance of a health sciences librarian (L.D.), keywords and search terms were chosen to address three concepts: (1) ACLR; (2) force plates; and (3) movement properties. The search strategy was developed as an extension of the search developed for Labban et al. (2024) whose search encompassed database inception to 13 March 2022 [21]. The search strategy was optimized for each of the nine databases: Medline, Embase, CINAHL, SPORTDiscus, Scopus, Web of Science, Proquest Dissertations and Theses, Science Direct, and Pubmed Central. For the current review, the same nine databases were searched from 2022 to 7 or 8 September 2024. The full search strategy is available in Appendix A.

### 2.5. Selecting Studies

Deduplication was performed in Covidence (Veritas Health Innovation, Melbourne, Australia) as well as manually in Excel to check for any overlapping studies from late 2021 and early 2022 that appeared in both the previous and current searches. Studies published in languages other than English, preprints, and conference abstracts were excluded. Title and abstract screening was performed to determine potentially relevant records followed by full-text review to determine final record selection, both of which were independently performed in Covidence by two reviewers (L.C., G.M.T.). All decisions and full-text exclusion reasons were recorded in Covidence.

Inclusion criteria were original or primary quantitative data studies with ACLR patients performing a CMJ or DJ on force plates no earlier than 6 months post-surgery with at least one kinetic parameter reported and the presence of a control group. Exclusion criteria included case reports or cross-sectional studies with no control group (studies with the contralateral limb serving as the control were also excluded), skeletally immature patients and measurements taken with non-force plate systems. Specific inclusion and exclusion criteria are reported in Table 1 with definitions of key terms outlined in Table 2.

### 2.6. Charting Data

After independent pilot extraction by two reviewers (L.C., G.M.T.), full-text data extraction was performed in Google Sheets by one reviewer (L.C.) and then verified by a second reviewer (G.M.T.). Discrepancies were discussed by the two reviewers. Data items included study and participant characteristics, date of study publication, country where study occurred, study outcome variables, and reported clearance criteria.

Although scoping review methodology does not require critical appraisal of included studies [26,27], Labban et al. (2024) [21] used the Modified Downs and Black (DB) checklist [29] with a 21-point scoring system for critical appraisal in their systematic review and meta-analysis; thus, the included studies from the current search for this scoping review were appraised with the same DB scoring to ensure consistency. Likewise, Labban et al. (2024) [21] identified the level of evidence of included studies using the Oxford Centre of Evidence-Based Medicine (OCEBM) 2011 model [30]; thus, the included studies from the current search for this scoping review were similarly categorized to ensure consistency. Discrepancies in DB scoring or OCEBM categorization were resolved by consensus between the two reviewers.

### 2.7. Reporting Results

For the purposes of this scoping review, the concept of clearance criteria is operationally defined as any reported requirement that is explicitly used to decide whether a research participant who has undergone ACLR is safe to perform study activities, particularly vertical jump testing. By this definition, these clearance criteria are as follows:

• Time from surgery (TFS) listed in either the introduction or methodology section in which a defined lower boundary of TFS is explicitly stated or specific testing time points after surgery are utilized.
• Functional or performance-based testing criteria (e.g., quadriceps strength LSI ≥ 90%, single leg horizontal hop LSI ≥ 85%).
• Medical evaluation (e.g., clearance from a healthcare professional).
• Explicit completion or participation in a rehabilitation program prior to entry into the study.

These clearance criteria are distinct from conventional inclusion or exclusion criteria which are general requirements used to select participants (e.g., sex, age, graft type) and would not influence the participants’ ability to safely complete the testing activities.

In order to detect patterns, the various clearance criteria were presented with information on jump type or jump type combinations.

## 3. Results

### 3.1. Identification of Studies

**Figure 1:**
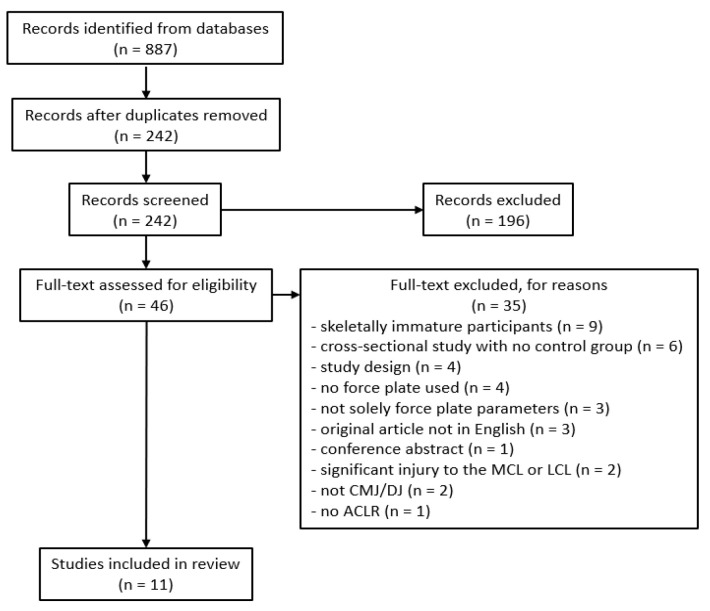
PRISMA diagram of study selection. MCL, medial collateral ligament; LCL, lateral collateral ligament; CMJ, countermovement jump; DJ, drop jump; ACLR, anterior cruciate ligament reconstruction.

A total of 887 records were identified from database searches, which was reduced to 242 after deduplication (Figure 1). Title and abstract screening reduced the records to 46, with 11 chosen for inclusion after full text review. These 11 studies were added to 24 studies from Labban et al. (2024) [21] which fit the same inclusion/exclusion criteria, for a total of 35 studies included in this review. The 24 studies from Labban et al. (2024) [21] had modified DB scores ranging from 8/21 to 12/21 and an OCEBM model level of evidence of 4. The 11 studies from the current search had modified DB scores ranging from 8/21 to 12/21 and an OCEBM model level of evidence of 4, except one where only the control arm of the randomized controlled trial was used, with an evidence level of 3 [31]. Consistent with the 24 studies from Labban et al. (2024) [21], common methodological limitations for the 11 included studies from the current search were incomplete descriptions of primary confounders, potential selection bias with studies not providing clear differentiation between those who chose to participate and those who did not, insufficient explanations of the validity of the methodological approaches used, and small sample sizes.

Papers evaluating the same cohort of participants but evaluating different aims, tasks evaluated, or outcomes were treated as a single study for the purpose of participant characteristics but treated independently for all other outcomes. Thus, 31 unique cohorts from the 35 included studies were used to report participant characteristics.

### 3.2. Study Characteristics

**Figure 2:**
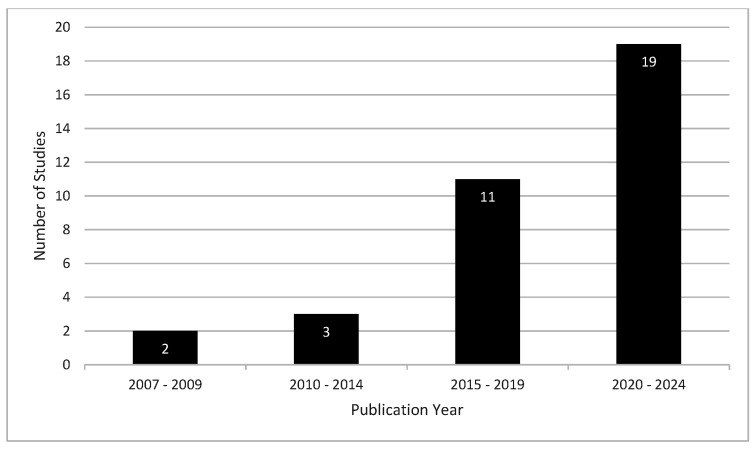
Number of studies by publication year grouped by date range.

The 35 included studies consisted of 27 (77.1%) cross-sectional studies with control groups [32,33,34,35,36,37,38,39,40,41,42,43,44,45,46,47,48,49,50,51,52,53,54,55,56,57,58] and 8 (22.9%) longitudinal studies [31,59,60,61,62,63,64,65]. Publication dates ranged from 2007 to 2024, with 19 of 35 (54.3%) studies published between 2020 and 2024 (Figure 2) [31,32,34,36,37,38,40,46,47,49,50,51,57,58,59,60,61,62,63]. Studies originated from 12 different countries: 13 (37.1%) studies from the USA [31,34,35,36,39,41,48,55,56,58,63,64,65], 6 (17.1%) from Qatar [46,47,51,57,61,62], 4 (11.4%) from Canada [43,44,49,60], 3 (8.6%) from Ireland [52,54,59], 2 (5.7%) from China [37,38], and 1 (2.9%) each from Brazil [33], Denmark [42], Germany [40], Iran [53], Portugal [32], Taiwan [50], and Turkey [45]. The types of jumps included also varied, with 13 (37.1%) studies using the double leg (DL) DJ [31,32,34,35,36,39,41,49,53,56,63,64,65], 5 (14.3%) using the single leg (SL) DJ [45,50,55,57,62], 6 (17.1%) using the DL CMJ [33,43,44,52,59,60], 3 (8.6%) with the SL CMJ [37,40,54], and 8 (22.9%) studies using combinations of the aforementioned jump types [38,42,46,47,48,51,58,61].

### 3.3. Participant Characteristics

Overall, 998 participants who underwent primary ACLR were included across 31 unique cohorts from the 35 included studies used to report participant characteristics, with 755 (75.7%) males, 169 (16.9%) females and 74 (7.4%) having no sex reported. Graft types used for the ACLR included 347 (34.8%) hamstring tendon grafts, 383 (38.4%) bone patellar tendon bone grafts and 268 (26.9%) grafts with either the type not reported or a less common graft type (i.e., posterior tibialis tendon). The youngest mean age reported for participants was 19.8 (±1.0) years [34,35,36] and the oldest was 36.93 (±8.19) years [32].

### 3.4. Clearance Criteria

Twenty-six of 35 (74.3%) studies reported a minimum TFS (Table 3) [31,32,33,34,35,36,37,38,40,41,42,45,48,49,50,51,52,54,57,58,59,60,62,63,64,65]. The earliest reported mean TFS at first assessment was 6.1 (±0.2) months [59] and the latest reported mean at 86.4 (±50.4) months post-ACLR [55]. Thirteen (37.1%) studies reported their mean TFS at first assessment between 6 and 9 months post-ACLR [46,47,51,52,53,54,59,60,61,62,63,64,65], four (11.4%) between 13 and 24 months [37,38,44,50], twelve (34.3%) at 24 or more months [31,32,33,34,35,36,40,41,42,55,56,58], and six studies (17.1%) that did not report specific time points of assessments post-ACLR [39,43,45,48,49,57].

Twenty-six (74.3%) studies reported some form of clearance criteria other than TFS to allow individuals to participate in the research activities (Table 3). Eighteen (51.4%) studies required individuals to have medical clearance from a healthcare professional [31,34,35,36,37,38,39,40,41,43,44,46,47,48,56,57,62], and 10 (28.6%) studies required participants to complete a rehabilitation program prior to beginning force plate jump testing [42,46,47,51,53,56,61,63,64,65]. Of the 18 studies that required medical clearance, 8 required clearance to be provided by a surgeon or physician [31,34,35,36,39,41,48,56], 3 required clearance from a physiotherapist or athletic therapist [50,57,62], 4 required clearance from both a surgeon and physiotherapist [37,38,46,47], and 3 did not specify what type of practitioner provided the medical clearance [40,43,44].

When analyzing the use of clearance criteria by jump type, jump types were listed in Table 3 from the most challenging to the least challenging: SL DJ, SL CMJ, DL DJ, DL CMJ. Two studies testing SL DJ in combination with other jumps required three clearance criteria: medical clearance, completion of a rehabilitation program and LSI [46,47]. Three studies testing SL DJ only required two clearance criteria, TFS and medical clearance [50,57,62], whereas a 2018 study used only TFS as clearance criteria for SJ DJ [45]. One 2008 study using a SL DJ did not report any criteria nor a TFS [55]. The three SL CMJ studies that used LSI clearance criterion additionally required TFS and medical clearance for a total of three clearance criteria (Table 3) [37,38,40]. All the remaining SL CMJ studies required only two or one clearance criteria: TFS, medical clearance and/or completed rehabilitation [42,51,54,58,61]. Likewise, all the DL DJ studies required either two or one clearance criteria: TFS, medical clearance and/or completed rehabilitation [31,32,34,35,36,39,41,48,49,53,56,63,64,65]. All the DL CMJ studies required only one clearance criterion, either TFS or medical clearance [33,43,44,52,59,60].

### 3.5. Limb Symmetry Indices as a Clearance Criteria

Five (14.3%) studies used LSI as a clearance criterion for participation in their study [37,38,40,46,47]. Among those 5 studies, 3 applied a SL horizontal hop for distance with a minimum LSI of ≥90%, ≥90%, and ≥85%, respectively [37,38,40]. The other two studies both employed a hop test battery requiring ≥90% symmetry along with quadriceps strength testing requiring ≥90% symmetry [46,47]. All five studies employing LSI clearance criteria had participants perform either a SL DJ or SL CMJ as part of jump testing. If LSI was one of the clearance criteria, then two other clearance criteria were also required (Table 3). All five studies were also published after 2022, required medical clearance for participation, and originated from either Qatar, China or Germany.

### 3.6. Limb Symmetry Indices as a Study Outcome

Beyond clearance criteria, LSI was more commonly reported as a study outcome, with 12 (34.3%) studies, including 3 of the studies that used LSI as a clearance criterion, also using LSI as part of their study outcomes [37,38,39,40,42,43,44,46,47,52,54,59]. Of the 12 studies that used LSI as a study outcome, a combination of LSIs derived from isokinetic or maximum voluntary isometric contraction strength testing [39,42,47,52,54,59], SL horizontal hop testing [40,47], force plate parameters [37,38,42,43,44,46,52,54,59], and joint moments and joint angles [37,38,42] were used.

## 4. Discussion

Across the 35 studies, four patterns emerged: (1) TFS remained a predominant clearance criteria; (2) TFS appeared to guide which additional criteria are used; (3) more demanding jump types tended to use a multi-criteria approach; and (4) LSI was infrequently used despite its popularity as a clearance criterion in RTP decision-making. Despite these patterns, no clear consensus on how to determine participant readiness for jump testing after ACLR emerged. Substantial heterogeneity with regard to timing of assessments after ACLR (<12 months vs. >24 months), level of sports participation, recruitment settings and jump task complexity made cross-study comparability challenging. As a result, the findings of this scoping review cannot provide concrete conclusions around best practices for clearance criteria. Nevertheless, the use and/or reporting of clearance criteria in rehabilitation settings can be extended to research design, especially in studies where the participants (a) have not yet been cleared for RTP, (b) are in the early stages of returning to their sport or (c) their rehabilitation was not elucidated.

The lack of common language and unclear separation of clearance versus inclusion criteria in some of the studies made it challenging to determine criteria intent, potentially obscuring key methodological differences between studies. For example, seven studies indicated a TFS range for study inclusion but did not make it clear if the lower boundary of the range was set for participant readiness or for the purpose of analyzing longitudinal outcomes such as changes in bone mineral density which occur over time after surgery [32,34,35,36,42,49,55]. One study provided only an upper TFS boundary, but did not report if a minimum was also used [39]. In these cases, a time-based threshold was presented without specifying its purpose. Thus, it must be acknowledged that this ambiguity may have led to contrived similarities between studies, and the TFS patterns synthesized here may not accurately represent author intention. Notwithstanding, these findings suggest that TFS continues to be viewed as a proxy measure of recovery status and readiness for dynamic testing, although it is now commonly accepted that TFS is an insufficient marker of recovery and should not be used in isolation [5,66].

Grouping studies by the mean TFS at first assessment helps to elucidate discernible patterns regarding criteria usage. Studies whose participants were less than 2 years post-surgery were more likely to report the need for participants to have completed or be in the process of completing a standardized rehabilitation program prior to entering the study [46,47,51,61,63,64,65]. On the other hand, studies whose participants were beyond 2 years post-surgery were more likely to report the requirement for medical clearance prior to study entry [31,34,35,36,39,40,41,43,44,48,56].

Requiring completion of a rehabilitation program would theoretically ensure that participants have undergone adequate rehabilitation progressions to safely perform the study activities. In 8 of 10 studies that required completion of a rehabilitation program, study participants were recruited from a sport medicine hospital/clinic, whereas no studies that recruited from the general community reported the need for completion of a rehabilitation program. This may suggest the feasibility of implementing rehabilitation clearance criteria influenced by the recruitment setting.

Using LSI as a clearance criterion was uncommon, with most studies instead reporting LSI as a study outcome. This may be in part due to the challenge of using LSI for screening purposes if the primary goal of the study is to evaluate limb symmetry. A potential approach to help navigate this challenge is to first apply an LSI clearance criterion using a low-risk test such as strength dynamometry or a horizontal hop test before clearing the patient to progress to more complex tasks such as force plate jumps where LSI can then be used as study outcomes. For the studies using LSI as a clearance criterion, the minimum required LSI ranged from 85 to 90%. In all five of these studies, medical clearance was required for study participation [37,38,40,46,47]. When LSIs were used as a study outcome, LSIs of less than 70% were seen for some force plate parameters [52] and less than 80% during knee strength testing [59]. These asymmetries were seen as late as 18 or more months after surgery, highlighting that neuromuscular deficits can still be present long after RTP clearance. One theoretical limitation of using an LSI of 85 to 90% is that it may be overly restrictive for controlled research activities and may result in a non-representative sample of how individuals perform after ACLR. A tiered screening approach may be helpful to avoid overly restrictive clearance criteria, where the required LSI for clearance is tightened as the inherent risk associated with study activities increases. Guidelines suggesting minimum clearance criteria LSI may also be warranted to help ensure participant safety without compromising study objectives. Finally, despite the popularity of LSI in both research and clinical practice, the authors of three studies suggest that LSI may provide a poor representation of function due to the deterioration of the healthy limb [50,51,57] and instead advocate that comparisons be made to healthy matched controls.

To determine if jump type affected selection of clearance criteria, we used the complexity of the jump task, with SL DJ being the most demanding and DL CMJ being the least [6]. DL landings can involve ground reaction forces up to 1.5 times body weight while SL jump tasks involve ground reaction forces anywhere from 2 to 6 times body weight, requiring increased strength and neuromuscular capacity to absorb force associated with the task [6]. These considerations were often reflected in this review, as the majority of SL jump studies required at least 1 other criterion other than TFS, typically implementing 2 to 3 clearance criteria whereas DL jump studies typically required only 1 or 2 criteria. In addition, the SL jump studies that utilized LSI criteria all required at least 2 additional criteria. Despite the heightened physical demands of SL jumps, only 2 of 15 SL jump studies reported strength testing as a clearance criteria [46,47]. In addition, three other SL jump studies required the completion of rehabilitation [42,51,61] and six others required medical clearance but did not specify if strength assessments were included in those other criteria [37,38,40,50,57,62].

Patterns of clearance criteria also emerged in the country of publication as those published in Qatar, USA and China appeared to frequently require either medical clearance or the completion of a specific rehabilitation program [31,34,35,36,37,38,39,41,46,48,51,56,57,61,62,63,64,65,67]. Conversely, the three studies from Ireland only reported minimum TFS and that the participant be a multidirectional field sport athlete [52,54,59]. These finding may suggest a relationship between different healthcare systems and how criteria are implemented and reported.

Finally, it is apparent that the reporting of safety-related elements including clearance criteria remains uncommon but is becoming more frequent in recently published studies. Another key research gap that remains is the underreporting of adverse events during testing. Although likely a rare occurrence, the lack of reporting makes it difficult to evaluate the safety of a study protocol if testing injuries are not disclosed. Standardized adverse event reporting during jump testing after ACLR, including the reporting of injury-free testing, would contribute to improved methodological rigor and clearance criteria selection. Further research could prioritize the evaluation and reporting of adverse event rates associated with testing in ACLR studies and perform prospective validation studies investigating clearance criteria protocols and safety outcomes. These results would inform the creation of consensus reporting standards and best practice guidelines for ACLR studies involving jump testing and other dynamic activities.

### 4.1. Clinical Implications

Currently there is no clear consensus on how to determine patient readiness to perform jump testing after ACLR. Clinically, it has been proposed that criteria such as a quadriceps LSI of 90% or greater on a knee extension test and leg press test, mastery of weighted single leg squats, mastery of change-of-direction drills, an LSI of 80% or great on hop test batteries, and adequate psychological readiness be achieved prior to initiating jumping [6,10,22,68]. Considering our current findings, a tiered screening approach may be warranted for ACLR jump studies, as jumps with increased physical demands (e.g., SL DJ) may necessitate more stringent criteria such as an LSI. This reviews evidence cannot inform best practice guidelines; however, adopting more rigorous standards may be advisable, pending future research. Additionally, more comprehensive reporting of clearance criteria by researchers, which will provide greater clarity around research participants’ physical status at the time of testing, may assist clinicians in generalizing study results to their own patients.

### 4.2. Limitations

Valuable insights are provided into the use of clearance criteria in force plate studies involving ACLR participants, although a few considerations should be kept in mind when interpreting the findings. We excluded three studies [69,70,71] that tested participants prior to 6 months post-surgery to align with current RTP recommendations for sport-specific training and dynamic testing [5,23,72,73] and limited included assessments to CMJs or DJs. Thus, our results may not generalize to other jump types or assessments performed within 6 months of surgery. Finally, as the focus of this review was to evaluate clearance criteria reporting, authors were not contacted for additional details, meaning these results reflect what was explicitly reported in the published manuscripts and may not represent what was actually done in practice.

## 5. Conclusions

This scoping review is the first focused examination of clearance criteria and limb symmetry indices in studies performing force plate-based jumping assessments with ACLR patients. The findings of this scoping review suggest that there is no clear consensus on how to determine participant readiness for jump testing post-ACLR. TFS, which is frequently cited, appears to often be supplemented or replaced with more stringent criteria dependent on the type of jump and the post-surgical time of testing. The clearance criteria considerations herein may be most relevant to studies that seek to recruit participants from the community or patients less than 2 years post-surgery when neuromuscular deficits may still persist. Future research is warranted to investigate the occurrences of ACLR re-injury during research activities to gain a deeper insight into the risk of jump testing in ACLR patients across the entire rehabilitation continuum. This would be facilitated by the reporting of adverse events becoming standard practice in ACLR research. The creation of best practice guidelines specifically for researchers and how clearance criteria usage influences rates of adverse events could also be of focus.

## Figures and Tables

**Table 1:** Inclusion and exclusion criteria from Labban et al. (2024) [21].

Inclusion Criteria	Exclusion Criteria
Human participants	Animal, cadaver, simulated or computer models
Original or primary quantitative data (cross-sectional with healthy control group, longitudinal with at least one kinetic force plate measurement at two different time points)	Not primary data (e.g., systematic review, literature review, meta-analysis, editorial, commentary, opinion papers or conference proceedings)
Primary ACLR with measurement taken at least 6 months post ACLR	Case report
At least one kinetic parameter measured solely by a force plate	Cross-sectional studies with no control group. Exclude if the control group is the contralateral limb
Participants performed drop jump or countermovement jump	Secondary ACLR (in ipsilateral or contralateral limb)
	Concomitant significant injuries or surgical interventions to the MCL or LCL
	Skeletally immature participants
	Congenital deformities
	Other musculoskeletal problems that could influence force plate parameters including foot disorders, hip disorders, and lower back and pelvic problems
	Neurological problems that could affect balance or neuromuscular co-ordinations
	ACL repair (not reconstruction) where the ACL was reattached
	Parameters measured with tools that do not employ force plate technologies (e.g., motion capture systems, isokinetic systems, contact mats)
	Kinetic parameters that cannot be measured with force plates solely (e.g., joint moments)
	Other types of jumps or other functional activities such as walking, running, squatting, cutting, pivoting, etc.)

**Table 2:** Definitions adapted from Labban et al. (2024) [21].

Term	Definition
Primary anterior cruciate ligament reconstruction (ACLR)	A first-time anterior cruciate ligament reconstruction, surgical tissue graft replacement of the anterior cruciate ligament to restore its function after injury
Skeletally immature	Participants under the age of 18 years old
Countermovement jump (CMJ)	From standing position, participant performs a downward motion to specific/self-selected depth before reversing the motion by triple-extending the hip, knee and ankle, jumping up for a maximum height
	
Drop jump (DJ)	Jumping/descending of a box placed behind a force plate, landing on the force plate and jumping vertically for a maximum height

**Table 3:** Clearance criteria sorted by jump type and date of publication.

Study/Year (Country)	TFS	Medical Clearance	Completed Rehabilitation	LSI	Jump Type
SL DJ or Combination					
Kotsifaki 2023 (Qatar) [46]		✔	✔	✔*	SL DJ SL CMJ DL DJ DL CMJ
Kotsifaki 2022 (Qatar) [47]		✔	✔	✔*	SL DJ SL CMJ
Lem 2022 (Taiwan) [50]	✔	✔			SL DJ
Read 2022 (Qatar) [62]	✔	✔			SL DJ
Read 2020 (Qatar) [57]	✔	✔			SL DJ
Kilic 2018 (Turkey) [45]	✔				SL DJ
Ortiz 2008 (USA) [55]					SL DJ
SL CMJ or Combination					
Chen 2024 (China) [37] Chen 2024 (China) [38]	✔	✔		✔*	SL CMJ DL CMJ
Maestroni 2023 (Qatar) [51]	✔		✔		SL CMJ DL CMJ
Maestroni 2023 (Qatar) [61]			✔		SL CMJ DL CMJ
Giesche 2022 (Germany) [40]	✔	✔		✔^^^	SL CMJ
Weidauer 2022 (USA) [58]	✔				SL CMJ DL CMJ
O’Malley 2018 (Ireland) [54]	✔				SL CMJ
Holsgaard-Larsen 2014 (Denmark) [42]	✔		✔		SL CMJ DL CMJ
DL DJ or Combination					
Andrade 2023 (Portugal) [32]	✔				DL DJ
Jeon 2022 (USA) [31]	✔	✔			DL DJ
Kuntze 2021 (Canada) [49]	✔				DL DJ
Chang 2020 (USA) [34] Huang 2020 (USA) [36] Chang 2018 (USA) [35]	✔	✔			DL DJ
Shimizu 2020 (USA) [63]	✔		✔		DL DJ
Krysak 2019 (USA) [48]	✔	✔			DL DJ DL CMJ
Shimizu 2019 (USA) [64] Shimizu 2019 (USA) [65]	✔		✔		DL DJ
Grooms 2018 (USA) [41]	✔	✔			DL DJ
Funk 2016 (USA) [39]		✔			DL DJ
Mohammadi 2012 (Iran) [53]			✔		DL DJ
Paterno 2007 (USA) [56]		✔	✔		DL DJ
DL CMJ or Combination					
Gagnon 2024 (Canada) [60]	✔				DL CMJ
Costley 2023 (Ireland) [59]	✔				DL CMJ
Miles 2019 (Ireland) [52]	✔				DL CMJ
Jordan 2018 (Canada) [43]		✔			DL CMJ
Jordan 2015 (Canada) [44]		✔			DL CMJ
Castanharo 2011 (Brazil) [33]	✔				DL CMJ

CMJ, countermovement jump; DJ, drop jump; DL, double leg; LSI, limb symmetry indices; SL, single leg; TFS, time from surgery. ^^^ minimum LSI of ≥85%, * minimum LSI of ≥90%.

## Data Availability

The data that supports the findings of this study are available from the corresponding author upon reasonable request.

## Abbreviations

The following abbreviations are used in this manuscript:

ACL	Anterior cruciate ligament
ACLR	Anterior cruciate ligament reconstruction
CMJ	Countermovement jump
DB	Modified Downs and Black
DJ	Drop jump
DL	Double leg
LSI	Limb symmetry indices
OCEBM	Oxford Centre of Evidence-Based Medicine
ROM	Range of motion
RTP	Return to play
SL	Single leg
TFS	Time from surgery

## Appendix A. Search Strategy as an Extension of the One Used by Labban et al. (2024) [21]

*Ovid MEDLINE(R) 2022—Current*
Date Searched: 7 September 2024; Result: 123

1. exp anterior cruciate ligament reconstruction/
2. ((Anterior cruciate ligament or ACL) adj8 (repair or reconstruct* or surgery or post-operativ* or postoperativ*)).mp.
3. 1 or 2
4. (((Bilateral or unilateral or countermovement or counter or squat or drop or one-leg* or two-leg* or single-leg* or double-leg*) adj4 jump*) or drop land* or jump landing or jump down or hop-test).mp.
5. (forceplate* or force plate* or force platform* or Kistler or GRF or GRFs or VGRF or VGRFs or pGRF? or ground reaction force* or kinetic* or center of pressure or centre of pressure or centre of mass or center of mass or Reactive strength index or RSImod or impulse or force-development or force-production or force time curve or (jump adj2 (height or duration or phase length)) or flight time or peak force* or limb-impulse* or phase specific or time curve or velocity or between limb difference* or between limb deficit* or ((Leg or legs or limb or limbs or knee or knees or functional or strength or muscle or index or indices or measur*) adj4 (asymmetr* or symmetr*))).mp.
6. 3 and 4 and 5

*Embase 2022 to 2024, 7 September 2024 (OVID Interface)*
Date Searched: 7 September 2024; Results: 134

1. anterior cruciate ligament reconstruction/
2. ((Anterior cruciate ligament or ACL) adj8 (repair or reconstruct* or surgery or post-operativ* or postoperativ*)).mp.
3. (forceplate* or force plate* or force platform* or Kistler or GRF or GRFs or VGRF or VGRFs or pGRF? or ground reaction force* or kinetic* or center of pressure or centre of pressure or centre of mass or center of mass or Reactive strength index or RSImod or impulse or force-development or force-production or force time curve or (jump adj2 (height or duration or phase length)) or flight time or peak force* or limb-impulse* or phase specific or time curve or velocity or between limb difference* or between limb deficit* or ((Leg or legs or limb or limbs or knee or knees or functional or strength or muscle or index or indices or measur*) adj4 (asymmetr* or symmetr*))).mp.
4. (((Bilateral or unilateral or countermovement or counter or squat or drop or vertical or one-leg* or two-leg* or single-leg* or double-leg*) adj4 jump*) or drop land* or jump landing or jump down or hop-test).mp.
5. (1 or 2) and 3 and 4
6. limit 5 to conference abstracts
7. 5 not 6

*CINAHL Plus with Full Text (EBSCOhost Interface) 2022–2024*
Date Searched: 7 September 2024; Results: 90

Deselecting: Apply equivalent subjects

((MH “Anterior Cruciate Ligament Reconstruction”) OR ((Anterior cruciate ligament or ACL) N8 (repair or reconstruct* or surgery or post-operativ* or postoperativ*))) AND (((Bilateral or unilateral or countermovement or counter or squat or drop or vertical or one-leg* or two-leg* or single-leg* or double-leg*) N4 jump*) or drop-land* or jump-landing or jump-down or hop-test) AND TX (forceplate* or force-plate* or force-platform* or Kistler or GRF or GRFs or VGRF or VGRFs or pGRF* or ground-reaction-force* or kinetic* or center-of-pressure or centre-of-pressure or centre-of-mass or center-of-mass or Reactive-strength-index or RSImod or impulse or force-development or force-production or force-time-curve or (jump adj2 (height or duration or phase length)) or flight-time or peak-force* or limb-impulse* or phase-specific or time-curve or velocity or between-limb-difference* or between-limb-deficit* or ((Leg or legs or limb or limbs or knee or knees or functional or strength or muscle or index or indices or measur*) N4 (asymmetr* or symmetr*)))

*SPORTDiscus with Full Text (EBSCOhost Interface) 2022–2024*
Date Searched: 8 September 2024; Results: 110

(((Anterior cruciate ligament or ACL) N8 (repair or reconstruct* or surgery or post-operativ* or postoperativ*))) AND (((Bilateral or unilateral or countermovement or counter or squat or drop or vertical or one-leg* or two-leg* or single-leg* or double-leg*) N4 jump*) or drop-land* or jump-landing or jump-down or hop-test) AND TX (forceplate* or force-plate* or force-platform* or Kistler or GRF or GRFs or VGRF or VGRFs or pGRF* or ground-reaction-force* or kinetic* or center-of-pressure or centre-of-pressure or centre-of-mass or center-of-mass or Reactive-strength-index or RSImod or impulse or force-development or force-production or force-time-curve or (jump N2 (height or duration or phase length)) or flight-time or peak-force* or limb-impulse* or phase-specific or time-curve or velocity or between-limb-difference* or between-limb-deficit* or ((Leg or legs or limb or limbs or knee or knees or functional or strength or muscle or index or indices or measur*) N4 (asymmetr* or symmetr*)))

*SCOPUS 2022–2024*
Date Searched: 8 September 2024; Results: 174

TITLE-ABS-KEY ((anterior-cruciate-ligament OR acl) W/8 (repair OR reconstruct* OR surgery OR post-operativ* OR postoperativ*)) AND TITLE-ABS-KEY (((bilateral OR unilateral OR countermovement OR counter OR squat OR drop OR vertical OR one-leg* OR two-leg* OR single-leg* OR double-leg*) W/4 jump*) OR drop-land* OR jump-landing OR jump-down or hop-test) AND TITLE-ABS-KEY (forceplate* OR force-plate* OR force-platform* OR kistler OR grf OR grfs OR vgrf OR vgrfs OR pgrf* OR ground-reaction-force* OR kinetic* OR center-of-pressure OR centre-of-pressure OR centre-of-mass OR center-of-mass OR reactive-strength-index OR rsimod OR impulse OR force-development OR force-production OR force-time-curve OR (jump W/2 (height OR duration OR phase-length)) OR flight-time OR peak-force* OR limb-impulse* OR phase-specific OR time-curve OR velocity OR between-limb-difference* OR between-limb-deficit* OR ((leg OR legs OR limb OR limbs OR knee OR knees OR functional OR strength OR muscle OR index OR indices OR measur*) W/4 (asymmetr* OR symmetr*)))

*Web of Science Core Collection (Indices = Science Citation Index (CI) Expanded, Social Sciences CI, Arts & Humanities CI, Emerging Sources CI) 2022–2024*
Date Searched: 8 September 2024; Results: 161

TS = ((anterior-cruciate-ligament OR acl) NEAR/8 (repair OR reconstruct* OR surgery OR post-operativ* OR postoperativ*)) AND TS=(((bilateral OR unilateral OR countermovement OR counter OR squat OR drop OR vertical OR one-leg* OR two-leg* OR single-leg* OR double-leg*) NEAR/4 jump*) OR drop-land* OR jump-landing OR jump-down or hop-test) AND TS = (forceplate* OR force-plate* OR force-platform* OR kistler OR grf OR grfs OR vgrf OR vgrfs OR pgrf* OR ground-reaction-force* OR kinetic* OR center-of-pressure OR centre-of-pressure OR centre-of-mass OR center-of-mass OR reactive-strength-index OR rsimod OR impulse OR force-development OR force-production OR force-time-curve OR (jump NEAR/2 (height OR duration OR phase-length)) OR flight-time OR peak-force* OR limb-impulse* OR phase-specific OR time-curve OR velocity OR between-limb-difference* OR between-limb-deficit* OR ((leg OR legs OR limb OR limbs OR knee OR knees OR functional OR strength OR muscle OR index OR indices OR measur*) NEAR/4 (asymmetr* OR symmetr*)))

*Proquest Dissertations and Theses Global (Web of Science) 2022–2024*
Date Searched: 8 September 2024; Results: 8

TS = ((anterior-cruciate-ligament OR acl) NEAR/8 (repair OR reconstruct* OR surgery OR post-operativ* OR postoperativ*)) AND TS=(((bilateral OR unilateral OR countermovement OR counter OR squat OR drop OR vertical OR one-leg* OR two-leg* OR single-leg* OR double-leg*) NEAR/4 jump*) OR drop-land* OR jump-landing OR jump-down or hop-test) AND TS = (forceplate* OR force-plate* OR force-platform* OR kistler OR grf OR grfs OR vgrf OR vgrfs OR pgrf* OR ground-reaction-force* OR kinetic* OR center-of-pressure OR centre-of-pressure OR centre-of-mass OR center-of-mass OR reactive-strength-index OR rsimod OR impulse OR force-development OR force-production OR force-time-curve OR (jump NEAR/2 (height OR duration OR phase-length)) OR flight-time OR peak-force* OR limb-impulse* OR phase-specific OR time-curve OR velocity OR between-limb-difference* OR between-limb-deficit* OR ((leg OR legs OR limb OR limbs OR knee OR knees OR functional OR strength OR muscle OR index OR indices OR measur*) NEAR/4 (asymmetr* OR symmetr*)))

*University of Alberta Science Direct Journals (Advanced Search) 2022–2024*
Date Searched: 8 September 2024; Results: 13

Do not include conference abstracts

Find articles with these terms: “ground reaction force” OR kinetic OR “asymmetry index” OR “symmetry index” OR “limb symmetry” OR “limb asymmetry” OR “leg symmetry” OR “Force plate” OR “jump height” Title, abstract or author-specified keywords: “Anterior Cruciate Ligament” AND (repair OR reconstruct OR reconstruction) AND (“drop jump” OR “vertical jump” OR “drop land” OR “landing task” OR “countermovement jump”)

*Pubmed Central 2022–2024*
Date Searched: 8 September 2024; Results: 74

((“anterior cruciate ligament”[Abstract] or ACL[Abstract]) AND (reconstruct[Abstract] OR reconstructed[Abstract] OR reconstruction[Abstract] OR repair[Abstract] OR repaired[Abstract] OR surgery[Abstract] OR post-operative[Abstract] OR postoperative[Abstract]) AND (vertical-jump[Abstract] OR drop-jump[Abstract] OR drop-land[Abstract] OR land-task[Abstract] OR single-leg-jump[Abstract] OR one-leg-jump[Abstract] OR double-leg-jump[Abstract] OR two-leg-jump[Abstract] OR countermovement-jump[Abstract] OR counter-jump[Abstract] OR vertical-jumps[Abstract] OR drop-jumps[Abstract] OR drop-landing[Abstract] OR landing-task[Abstract] OR landing-tasks[Abstract] OR single-leg-jumps[Abstract] OR one-leg-jumps[Abstract] OR double-leg-jumps[Abstract] OR two-leg-jumps[Abstract] OR countermovement-jumps[Abstract] OR counter-jumps[Abstract])) AND (“ground reaction force” OR “ground reaction forces” OR “force plate” OR “force plates” OR forceplate* OR kinetic OR kinetics OR “asymmetry index” OR “symmetry index” OR “asymmetry indices” OR “symmetry indices” OR “limb symmetr*” OR “limb asymmetr*” OR “leg symmetr*” OR between-limb-difference* OR between-limb-deficit* OR “jump height” OR “Peak force” OR “force development” OR force-production OR force-time-curve OR kistler OR grf OR grfs OR vgrf OR vgrfs OR pgrf* OR reactive-strength-index OR rsimod OR center-of-pressure OR centre-of-pressure OR centre-of-mass OR center-of-mass OR reactive-strength-index OR rsimod OR “concentric impulse” or “eccentric impulse” or “unweighting impulse” or “breaking impulse” or “deceleration impulse” OR limb-impulse* OR “flight time”)
